# Subcloning and characterization of highly metastatic cells derived from human esophageal squamous cell carcinoma KYSE150 cells by *in vivo* selection

**DOI:** 10.18632/oncotarget.16668

**Published:** 2017-03-29

**Authors:** Masafumi Okuda, Jun Inoue, Naoto Fujiwara, Tatsuyuki Kawano, Johji Inazawa

**Affiliations:** ^1^ Department of Molecular Cytogenetics, Medical Research Institute and Graduate School of Medical and Dental Sciences, Tokyo Medical and Dental University, Tokyo, Japan; ^2^ Department of Gastrointestinal Surgery, Graduate School of Medical and Dental Sciences, Tokyo Medical and Dental University, Tokyo, Japan; ^3^ Bioresource Research Center, Research and Industry-University Alliance Organization, Tokyo Medical and Dental University, Tokyo, Japan

**Keywords:** lung metastasis, esophageal squamous cell carcinoma (ESCC), in vivo selection, cytokine, inflammation

## Abstract

Esophageal cancer is the eighth most common cancer and the sixth most common cause of cancer-related deaths worldwide. Despite the research progress in understanding the disease, the mechanism underlying the metastasis is still unclear. Here, we successfully generated a highly metastatic cell subline, designated as KYSE150-LuM, derived from an esophageal squamous cell carcinoma cell line (KYSE150) by *in vivo* selection. To elucidate the mechanisms driving metastasis, we characterized the gene expression differences between LuM cells and parent KYSE150 cells. IL-6, IL-1β, and LCN2, previously associated with tumor growth and metastasis, were up-regulated in LuM cells. Recent studies on cancer have increasingly focused on the tumor microenvironment, from which these cytokines are released. The fact that these three cytokines (IL-6, IL-1β, LCN2) were up-regulated in LuM cells indicates that these highly metastatic cells obtained through *in vivo* selection will be a useful resource for further studies on elucidating the mechanisms underlying the tumor microenvironment which is associated with cytokine-related tumor growth and metastasis. Moreover, LuM cells could disseminate to the lung in shorter period of time *in vivo*, indicating their utility for *in vivo* experiments of metastasis and new therapeutic targets in a shorter period of time than currently possible.

## INTRODUCTION

Esophageal cancer is the eighth most common cancer and the sixth most common cause of cancer-related deaths worldwide [1]. There are two major histological types of esophageal cancer: squamous cell carcinoma and adenocarcinoma. In Asia, the most common type is esophageal squamous cell carcinoma (ESCC), which has been associated with environmental factors such as chronic smoking, alcohol consumption, and infection [2, 3]. Moreover, several genes such as *ADH1B*, *ALDH2* [4], and *YAP* [5] have been identified to play a role in ESCC risk and development. Despite the research progress in understanding the disease and advances in therapeutic strategies, the 5-year relative survival rate of patients with ESCC with distant metastasis remains low at only 4.3% [6]. Rapid progression, local recurrence, and distant metastasis are the main reasons for the low survival rate. However, the mechanism underlying the metastasis of esophageal cancer is still unclear.

The process of metastasis consists of multiple and sequential steps, including proliferation, angiogenesis, motility, and invasion [7]. The outcome of metastasis depends on various types of interactions between metastatic tumor cells and a multitude of host factors [7, 8]. To investigate the mechanisms of cancer metastasis, our laboratory has used *in vivo* selection [9], which is a commonly used method to select highly metastatic variants [8, 9]. We considered that detection of metastasis by *in vivo* selection could reflect the ability of tumor cells to survive in the circulation and grow in a distant organ. Therefore, the characterization of highly metastatic variants that were selected by *in vivo* selection may improve our understanding of the mechanisms driving cancer metastasis.

In the present study, we selected a highly metastatic ESCC subline, designated as KYSE150-LuM (hereinafter, LuM), derived from parent ESCC cell line, KYSE150 [10], by *in vivo* selection. To help elucidate the mechanisms driving metastasis, we characterized the gene expression differences between the highly metastatic cells and parent cells. We particularly focused on the expression and secretion of cytokines known to be involved in tumor development and metastasis between LuM cells and parent KYSE150 cells. This work is expected to provide a new resource for more detailed studies on metastasis in ESCC, facilitating the development of new therapeutic targets for pre-clinical and clinical trials.

## RESULTS

### Generation of highly metastatic cells derived from the ESCC KYSE150 cell line

**Figure 1:**
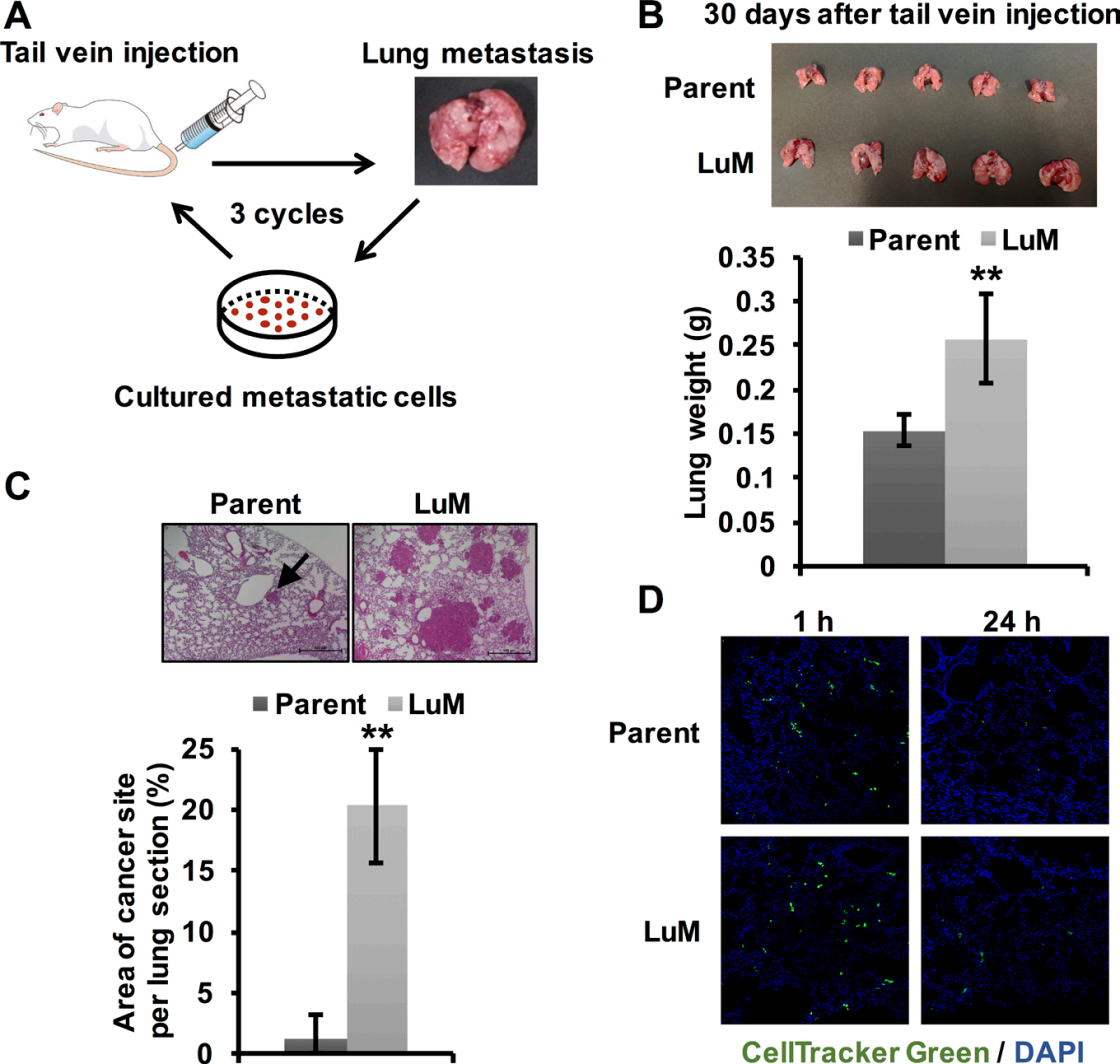
Generation of highly metastatic cells derived from the KYSE150 cell line (**A**) The generation of highly metastatic cells derived from KYSE150, an ESCC cell line, by *in vivo* selection during the process of lung metastasis. The selection cycle was repeated three times. (**B**) *Ex vivo* mouse lung images and weights at 30 days after tail vein injection of parent KYSE150 cells and LuM cells (5.0 × 10^5^ cells in 100 μl of PBS, 5 mice, respectively). Data are represented as mean ± standard deviations. **p < 0.01. (**C**) Hematoxylin and eosin-stained lung sections and quantification of lung metastatic foci at 30 days after tail vein injection of parent KYSE150 cells and LuM cells (5.0 × 10^5^ cells in 100 μl of PBS, 5 mice, respectively). Scale bar, 500 μm. The arrow indicates metastatic foci. Data are represented as mean ± standard deviations. **p < 0.01. (**D**) Extravasation assay using immunofluorescent analysis. Cells labeled with CellTracker™ Green were injected into the tail veins of the mice. After a defined time, the mice were sacrificed and frozen sections of the lung were prepared.

To identify candidate metastasis-related genes in ESCC, we first established the highly metastatic cell subline by *in vivo* selection [9]. Luciferase-labeled KYSE150 cells were injected into the tail veins of mice. After lung metastasis was observed, the metastatic nodules were cultured. After repeating this process of inducing lung metastasis three times, we successfully established the highly metastatic KYSE150 subline, designated as LuM (Figure 1A).

To confirm whether LuM cells had increased metastatic ability, we injected the selected cells into the tail veins of the mice. LuM cells disseminated to the lung approximately 30 days earlier than observed for parent KYSE150 cells, which usually take approximately 90 days to disseminate to the lung (Figure 1B). Moreover, the lung size and weight were higher in mice injected with LuM cells than with parent KYSE150 cells. Moreover, in hematoxylin and eosin-stained sections, the area of metastatic foci was significantly higher in the mice that received injections of LuM cells than in those injected with parent KYSE150 cells (Figure 1C). On the other hand, there were no significant differences in the frequency of extravasation to the lung between the two groups within 24 h after tail vein injection (Figure 1D), suggesting that the ability related with migration, invasion, and survival to adapt within tumor microenvironment in lung, rather than extravasation ability, may be up-regulated in LuM cells.

### Characterization of the highly metastatic cell line LuM

**Figure 2:**
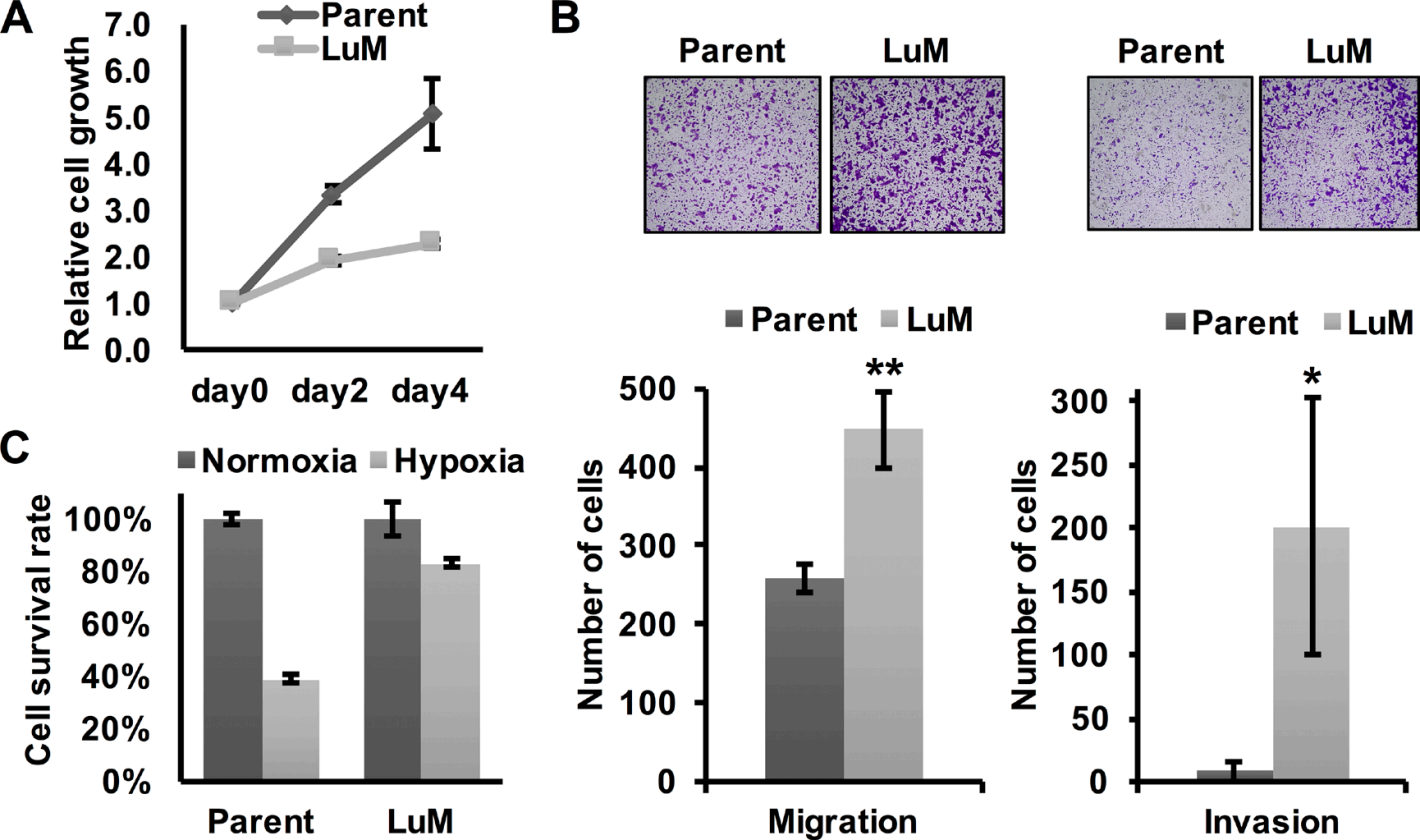
Characterization of the highly metastatic cell line LuM (**A**) Cell growth curves were determined by crystal violet staining. Cell growth was slower in LuM cells compared with that of the parent KYSE150 cells *in vitro*. Data are represented as mean ± standard deviations. (**B**) Cell motility and invasion was assessed by a transwell migration and invasion assay with parent KYSE150 cells or LuM cells. Data are represented as mean ± standard deviations. *p < 0.05. **p < 0.01. (**C**) Cell survival rates were determined by crystal violet staining. Cell viability under hypoxic conditions (O_2_ < 0.1%, 48 h) was higher in LuM cells compared with that of parent KYSE150 cells *in vitro*. Data are represented as mean ± standard deviations.

To investigate the differential characteristics between parent KYSE150 cells and LuM cells, we first performed *in vitro* experiments. Although the metastatic ability was clearly higher in LuM cells than parent KYSE150 cells *in vivo*, cell growth was slower in LuM cells *in vitro* (Figure 2A). However, the *in vitro* migration and invasion ability were higher in LuM cells (Figure 2B). Interestingly, under hypoxic conditions, the cell viability of LuM cells was higher than that of parent KYSE150 cells (Figure 2C).

### Involvement of cytokines in the enhanced metastatic ability of LuM cells

**Figure 3:**
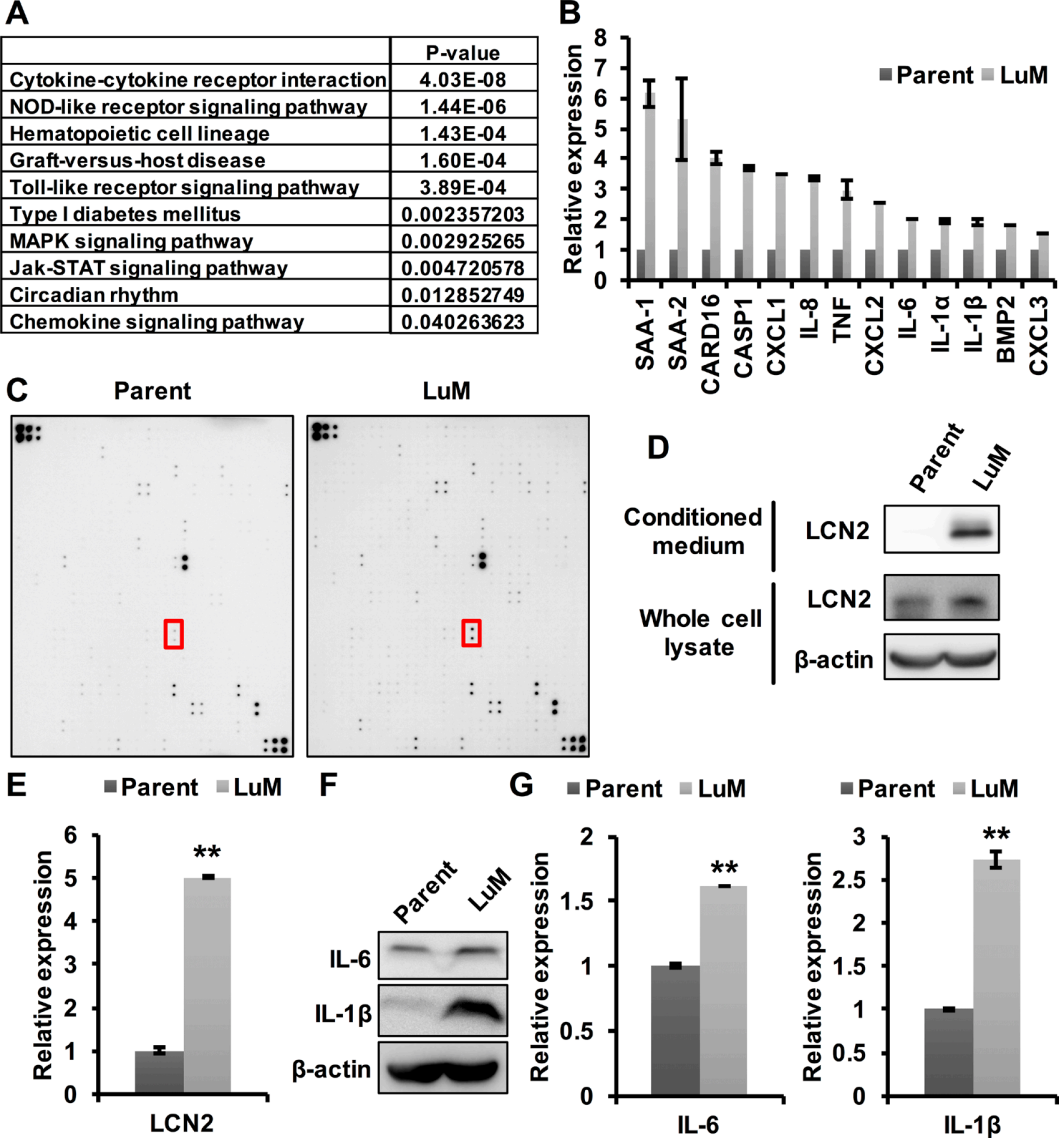
Involvement of cytokines in the enhanced metastatic ability of LuM cells (**A**) Strategy for identifying metastasis-associated genes. In the expression array analysis, 246 genes showed increased expression levels in LuM cells compared with parent KYSE150 cells. Subsequently, gene pathway enrichment analysis showed a strong association of the candidate genes with the cytokine-cytokine receptor pathways. (**B**) Expression levels of candidate genes by qRT-PCR. Error bars are standard deviations of the means of duplicate experiments. (**C**) Cytokine array analysis of the cell culture supernatant from parent KYSE150 cells or LuM cells. The red box indicates LCN2. Upper left and lower right on both membrane indicate reference spot. Information of each position on array were shown in manufacturer's instructions. (**D**) LCN2 protein expression in conditioned medium and the whole cell lysate by western blotting in parent KYSE150 cells and LuM cells. (**E**) LCN2 secretion levels of the supernatant from parent KYSE150 cells and LuM cells by ELISA. Data are represented as mean ± standard deviations. **p < 0.01. (**F**) IL-6 and IL-1β protein expression in the whole cell lysate by western blotting in parent KYSE150 cells and LuM cells. (**G**) IL-6 and IL-1β secretion levels of the supernatant from parent KYSE150 cells and LuM cells by ELISA. Error bars are standard deviations of the means of duplicate experiments. Data are represented as mean ± standard deviations. **p < 0.01.

Previous studies have shown that cytokines play a key role in tumor growth and metastasis in the tumor microenvironment (TME) [11]. Therefore, we hypothesized that the enhanced metastasis of LuM cells may be associated with several cytokines, which was tested through expression array analysis between parent KYSE150 cells and LuM cells. The expression levels of 246 genes were found to be up-regulated by more than 3-fold in LuM cells compared with parent KYSE150 cells (Supplementary Table 2). Furthermore, gene pathway analysis in the KEGG database for these 246 genes revealed that the cytokine-cytokine receptor interaction pathway was significantly up-regulated in LuM cells (Figure 3A).

To examine the specific cytokines associated with LuM cells, the differential expression levels of a subset of the up-regulated genes were evaluated with qRT-PCR, which confirmed increased expression levels of several of the genes, including cytokines, in LuM cells (Figure 3B). Cytokine array analysis between parent KYSE150 cells and LuM cells demonstrated that several cytokines were up-regulated in the supernatant of LuM cells compared with that of parent KYSE150 cells (Figure 3C, Table 1). Among these up-regulated proteins, we focused on LCN2, since it was previously shown to promote the migration and invasion of ESCC cells [12]. The expression level of LCN2 protein was higher in both the cell lysate and the supernatant of LuM cells than in those of parent KYSE150 cells (Figure 3D and 3E). In addition, siRNA-mediated inhibition of LCN2 resulted in the reduced migration and invasion abilities in LuM cells (Supplementary Figure 1).

**Table 1:** List of up-regulated secreted proteins by cytokine array analysis

No.	gene	Location	fold change
**1**	IL-17 R	3p25	32.6
**2**	EGF-R/ErbB1	7p12	20.1
**3**	GREMLIN	15q13.3	17.8
**4**	IGFBP-2	2q35	17.1
**5**	GDF-15	19p13.11	15.7
**6**	IL-7	8q12	14.7
**7**	Lipocalin-2	9q34	11.8
**8**	LBP	20q11.23	6.8
**9**	IL-15R alpha	10p15.1	6.0
**10**	VEGF	6p12	5.9
**11**	sFRP-4	7q14.1	4.6
**12**	BMP2	20p12	2.9
**13**	S100A10	1q21	2.1
**14**	IGFBP−7	4q12	1.9

Furthermore, the cytokines IL-6 and IL-1β, previously associated with tumor growth and metastasis [13–16], were also up-regulated in both the cell lysate and the supernatant of LuM cells (Figure 3F and 3G), confirming the results of the expression array analysis.

Taken together, these results suggest that the highly metastatic ability of LuM cells may be explained by the interaction of these cytokines.

## DISCUSSION

In the present study, we successfully generated a highly metastatic cell line, designated as LuM, derived from parent ESCC cell line, KYSE150 [10], by *in vivo* selection. The selection of highly metastatic populations points to substantial tumor heterogeneity [8]. LuM cells could disseminate to the lung within approximately 30 days, indicating their utility for *in vivo* experiments of metastasis and new therapeutic targets in a shorter period of time than currently possible.

The characteristics of these highly metastatic cells suggest the involvement of several cytokines in metastasis. The up-regulated cytokines are mainly associated with inflammation. Indeed, the constitutive activation of inflammatory pathways is known to induce the development of esophageal cancer [14]. Inflammation activates the transcription of downstream genes and the activities of enzymes associated with tumor growth and survival [11].

In particular, the IL-6/signal transducer and activator of transcription 3 (STAT3) pathway and IL-1β are well known to be involved in inflammation-related pathways that play a key role in carcinogenesis [13, 15, 17]. Several studies have demonstrated increased expression levels of IL-6, STAT3, and IL-1β both *in vitro* and in ESCC patients [16, 18, 19]. IL-6 is a cytokine that triggers the downstream activation of STAT3 transcription factors [20]. Normally, the IL-6/STAT3 pathway serves to prevent apoptosis in cells, which enables their survival in toxic inflammatory environments. However, this pathway can also prevent apoptosis in tumors, thereby promoting tumor growth, survival, and metastasis [21]. Moreover, IL-1β is a pro-inflammatory cytokine that promotes tumor invasiveness and metastasis [17]. The expression of IL-1β was found to be higher in ESCC samples than in normal tissues and correlated with a poor prognosis [16]. In addition, LCN2, which is involved in the cell differentiation and tumor invasion of ESCC [22], is up-regulated by IL-1β [23].

The fact that these three cytokines (IL-6, IL-1β, LCN2) were up-regulated in LuM cells indicates that these highly metastatic cells obtained through *in vivo* selection will be a useful resource for further studies on elucidating the mechanisms underlying cytokine-related tumor growth and metastasis. In the present study, in order to reveal the mechanism of over-expression of LCN2 and other genes up-regulated in LuM cells compared with parent KYSE150 cells, we performed a subtractive array-CGH analysis between LuM cells and parent KYSE150 cells using array-CGH system. However, any specific copy number aberrations between LuM cells and parent KYSE150 cells could not be detected (data not shown).

We consider that this highly metastatic subline is useful resource for further study including comparative expression analysis with clinical samples due to understand a possible mechanism driving metastatic property and to develop therapeutic strategy against metastatic ESCC cells. Furthermore, recent studies on cancer have increasingly focused on the tumor microenvironment (TME), from which these cytokines are released. Several other components of the TME have also been associated with tumor growth and metastasis, such as VEGF, PD-1, and CTLA-4, and treatments targeting these TME factors are currently being explored in clinical trials [11]. However, the detailed characteristics of the TME have not yet been fully explained. Thus, further characterization of highly metastatic cells obtained through *in vivo* selection may help to elucidate the characteristics and functions of the TME. It is expected that further studies of the TME may lead to the development of novel therapeutic targets.

## MATERIALS AND METHODS

### Cell culture

The KYSE150 cell line was kindly provided by Dr. Y. Shimada (Kyoto University, Japan) and was maintained in Roswell Park Memorial Institute (RPMI) 1640 medium containing 10% fetal bovine serum (FBS). KYSE150 cells were maintained at 37°C with 5% CO_2_.

### Mice, *in vivo* selection, *in vivo* metastasis assay, and bioluminescence imaging

NOD/SCID mice were purchased from the Charles River Laboratories Japan, and animal experiments were performed according to the institutional animal ethics guidelines. To establish the highly metastatic subline by *in vivo* selection, luciferase-labeled KYSE150 cells (5.0 × 10^5^ cells in 100 μl of phosphate-buffered saline [PBS]) were injected into the tail vein of the mice. For bioluminescent observation of lung metastasis, the mice were injected intraperitoneally with 150 mg/kg d-luciferin potassium salt (SYNCHEM UG & Co. KG; Felsberg/Altenburg, Germany) and observed with the luminometer (Photon IMAGER RT; BIOSPACE LAB, Paris, France). Upon detection of lung metastasis, the mice were sacrificed and the metastatic nodules were collected, cut into small pieces, and incubated in dissociation solution (Dispase^®^ II; Roche, Mannheim, Germany) with the cells. The cells obtained from the metastasized lung tissues were cultured in a culture dish as described above. After repeating this process three times, the highly metastatic subline, designated as LuM, was established. Short tandem repeat analysis was conducted to confirm that the LuM cells were indeed derived from KYSE150 cells.

### Gene expression array analysis

The Agilent 8 × 60 K array (Agilent Technologies, CA, USA) was used for differential gene expression analysis between parent KYSE150 cells and LuM cells according to the manufacturer's instructions. The raw data were analyzed with GeneSpring GX10 software (Agilent Technologies). After selection, candidate genes associated with metastasis were subjected to Kyoto Encyclopedia of Genes and Genomes (KEGG) pathway analysis to determine the pathways that were enriched.

### Cell growth and cell survival assay

Cell survival was assessed by the crystal violet (CV) staining assay. Cells were washed in PBS and fixed with 0.1% CV in 10% formaldehyde in PBS for 10 min. After excess CV solution was discarded, the stained cells were completely air-dried, and then lysed with a 2% sodium dodecyl sulfate (SDS) solution with shaking for 2 h. Optical density (OD) values of the absorbance were measured at 560 nm using a microplate reader (ARVOmx; Perkin-Elmer, MA, USA), and the percent absorbance of each well was determined. The OD absorbance values of cells in control wells were arbitrarily set at 100% to determine the percentage of viable cells.

### Migration and invasion assays

Transwell migration and invasion assays were carried out in 24-well modified chambers that were not pre-coated (migration assay) or pre-coated (invasion assay) with Matrigel (Corning BioCoat; Corning Incorporated, NY, USA). Cells in serum-free medium were transferred into the upper chamber. After 48-h incubation, the migrated cells in the lower chamber with 10% FBS as a chemoattractant were fixed and stained with 0.1% CV in 10% formaldehyde and counted with ImageJ software (National Institutes of Health, MD, USA). Each assay was performed in triplicate.

### Western blot analysis

Whole cell lysates were subjected to SDS-polyacrylamide gel electrophoresis, and proteins were transferred to polyvinylidene fluoride membranes (GE Healthcare). After blocking with Tris-buffered saline containing 0.05% Tween-20 and 5% non-fat dry milk for 1 h, the membrane was reacted with the primary antibody for interleukin (IL)-6 (ab6672; Abcam plc, Cambridge, UK; 1:1,000 dilution), beta-actin (A5441; Sigma-Aldrich, MO, USA; 1:5,000 dilution), IL-1β (AB-201-NA; R&D Systems, MN, USA; 1:1,000 dilution), and lipocalin-2/NGAL (LCN2; MAB1757; R&D Systems, 1:1,000 dilution). The membrane was washed and exposed to horseradish peroxidase-conjugated anti-mouse or -rabbit IgG antibodies (both diluted at 1:5,000) for 2 h. The bound antibodies were visualized on the LAS3000 imaging system (FUJIFILM, Tokyo, Japan) using a Pierce ECL Western detection kit according to the manufacturer's instructions (Thermo Scientific, MA, USA).

### Quantitative reverse transcription-polymerase chain reaction (qRT-PCR)

qRT-PCR was performed using an ABI PRISM 7500 Fast Real Time PCR System with the KAPA SYBR Fast qPCR kit (Kapa Biosystems, MA, USA) according to the manufacturer's instructions. Primers were indicated in Supplementary Table 1. Gene expression values were calculated as ratios based on the differences in cycle threshold values between the genes of interest and an internal reference gene (GAPDH) that provides a normalization factor for the amount of RNA isolated from a specimen. The values were subsequently normalized with the control values to determine the relative expression levels. Each assay was performed in duplicate for each sample.

### Enzyme-linked immunosorbent assay (ELISA)

Cell culture supernatants were collected and analyzed by ELISA using a Quantikine^®^ ELISA kit (R&D Systems) according to the manufacturer›s instructions.

### Cytokine antibody array analysis

The expression levels of human cytokines in the cell culture supernatants, including IL-1β and LCN2, were measured by antibody array (Label-Based (L-Series) Human Antibody Array 507 (L-507) Membrane Kit; RayBiotech, GA, USA) according to the manufacturer's instructions. Information of each position on array were shown in manufacturer's instructions.

### Transfection of small interference RNA (siRNA)

The siRNA for LCN2 (siGENOME SMARTpool; M-003679-02-0005) and control non-specific siRNA (siGENOME Non-targeting Pool; D-001810-05) were obtained from Thermo Scientific. The siRNA was transfected individually into cells at the indicated concentration using Lipofectamine RNAiMAX (Invitrogen) according to the manufacturer's instructions.

### Statistical analysis

Differences between subgroups (LuM cells and parent KYSE150 cells) were tested by the Student *t-test*. A *P-value* of < 0.05 was considered statistically significant.

## SUPPLEMENTARY MATERIALS FIGURES AND TABLES




