# Supplementary material for: Subcloning and characterization of highly metastatic cells derived from human esophageal squamous cell carcinoma KYSE150 cells by in vivo selection
Source: Oncotarget. 2017 Mar 29;8(21):34670–7. doi: 10.18632/oncotarget.16668 (PMC5471001; doi:10.18632/oncotarget.16668)
Supplement: Supplementary file 1 [file oncotarget-08-34670-s001.pdf]

Subcloning and characterization of highly metastatic cells derived from human esophageal squamous cell carcinoma KYSE150 cells by *in vivo* selection

Supplementary Materials

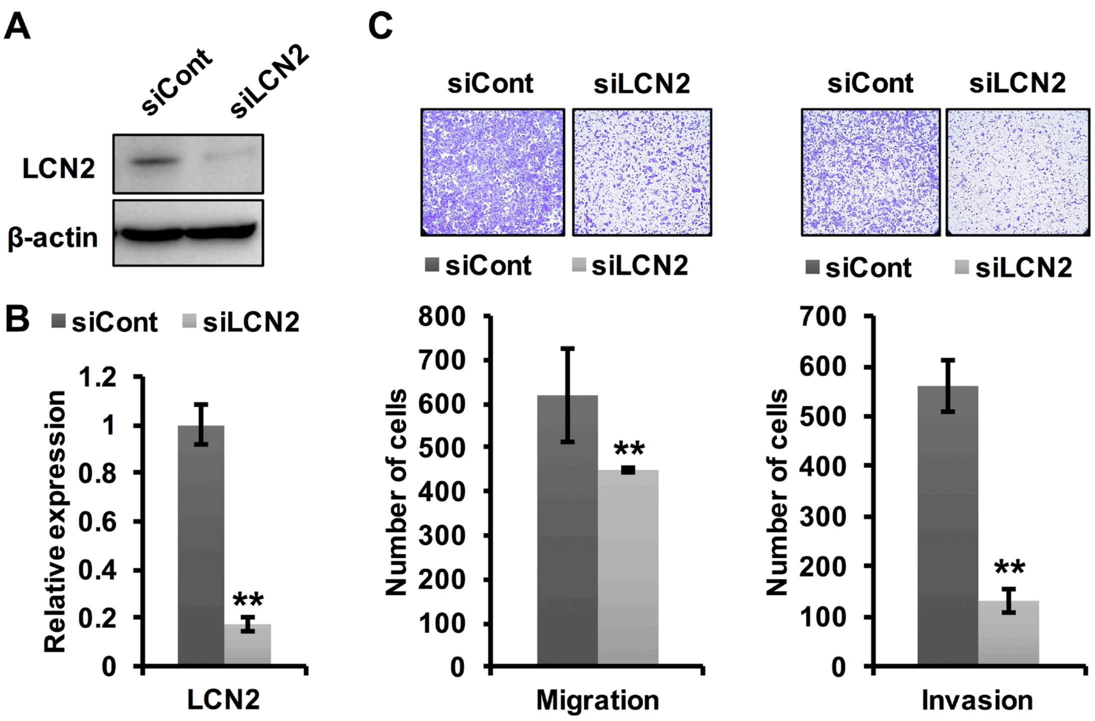

Supplementary Figure 1: siRNA-mediated knockdown of LCN2. (**A**) LCN2 protein expression in the whole cell lysate by western blotting in LuM cells which transfected siCont (siRNA control) or siLCN2 (siRNA for LCN2). (**B**) LCN2 secretion levels of the supernatant from siRNA transfected LuM cells by ELISA. Data are represented as mean ± standard deviations. ***p* < 0.01. (**C**) Cell migration and invasion was assessed by a transwell migration and invasion assay with siRNA transfected LuM cells. Data are represented as mean ± standard deviations. ***p* < 0.01.

Supplementary Table 1: Sequence of primers for qRT-PCR

Name	Forward primer (5′ to 3′)	Reverse primer (5′ to 3′)
SAA-1	ATTGTGTACCCTCTCCCC	CTGCAGAAGTGATCAGCA
SAA-2	ATTATATGCATTATCTCAGC	AGCCCATGTTGTAGCAAACC
CARD16	TTCCATGGGTGAAGGTACAA	AGCTTGATTCTGCCTTCTGG
CASP1	TGCCTGTTCCTGTGATGTGG	TGTCCTGGGAAGAGGTAGAAACATC
CXCL1	AACCGAAGTCATAGCCACAC	GTTGGATTTGTCACTGTTCAGC
IL-8	AGGTGCAGTTTTGCCAAGGA	TTTCTGTGTTGGCGCAGTGT
TNF	AGCCCATGTTGTAGCAAACC	TGAGGTACAGGCCCTCTGAT
CXCL2	TGCAGGGAATTCACCTCAAG	TGAGACAAGCTTTCTGCCCA
IL-6	GAGGCACTGGCAGAAAAAA	TGGCATTTGTGGTTGGGTCA
IL-1α	ATCCTGAATGACGCCCTCAA	TGGATGGGCAACTGATGTGA
IL-1β	GGACAAGCTGAGGAAGATGC	TCGTTATCCCATGTGTCGAA
BMP2	ATGGATTCGTGGTGGAAGTG	GTGGAGTTCAGATGATCAGC
CXCL3	GGAGCACCAACTGACAGGAG	TAAGGGCAGGGACCACC

Supplementary Table 2: List of the 246 genes which up-regulated by more than 3-fold in LuM cells compared with parent KYSE150 cells. See Supplementary_Table_2
