# Supplementary material for: Subcloning and characterization of highly metastatic cells derived from human esophageal squamous cell carcinoma KYSE150 cells by in vivo selection
Source: Oncotarget. 2017 Mar 29;8(21):34670–7. doi: 10.18632/oncotarget.16668 (PMC5471001; doi:10.18632/oncotarget.16668)
Supplement: Supplementary file 2 [file oncotarget-08-34670-s002.docx]

| **Supplementary Table 2. List of the 246 genes which up-regulated by more than 3-fold in LuM cells compared with parent KYSE150 cells.** | | |
| --- | --- | --- |
| Fold change | GeneSymbol | Description |
| 56.78021 | CASP1 | Homo sapiens caspase 1, apoptosis-related cysteine peptidase (CASP1), transcript variant alpha, mRNA [NM_033292] |
| 38.087242 | IL24 | Homo sapiens interleukin 24 (IL24), transcript variant 3, mRNA [NM_001185156] |
| 33.019363 | KRT6B | Homo sapiens keratin 6B (KRT6B), mRNA [NM_005555] |
| 32.595036 | S100A9 | Homo sapiens S100 calcium binding protein A9 (S100A9), mRNA [NM_002965] |
| 32.510532 | SAA2 | Homo sapiens serum amyloid A2 (SAA2), transcript variant 1, mRNA [NM_030754] |
| 31.159483 | SPRR1A | Homo sapiens small proline-rich protein 1A (SPRR1A), transcript variant 2, mRNA [NM_005987] |
| 30.82858 | LGALS7 | Homo sapiens lectin, galactoside-binding, soluble, 7 (LGALS7), mRNA [NM_002307] |
| 26.072186 | S100A8 | Homo sapiens S100 calcium binding protein A8 (S100A8), mRNA [NM_002964] |
| 25.56192 | SCCPDH | Homo sapiens saccharopine dehydrogenase (putative) (SCCPDH), mRNA [NM_016002] |
| 24.600895 | BST2 | Homo sapiens bone marrow stromal cell antigen 2 (BST2), mRNA [NM_004335] |
| 22.733986 | SAA1 | Homo sapiens serum amyloid A1 (SAA1), transcript variant 1, mRNA [NM_000331] |
| 22.22008 | IGFBP2 | Homo sapiens insulin-like growth factor binding protein 2, 36kDa (IGFBP2), mRNA [NM_000597] |
| 20.74937 | NUPR1 | Homo sapiens nuclear protein, transcriptional regulator, 1 (NUPR1), transcript variant 1, mRNA [NM_001042483] |
| 20.38691 | LCN2 | Homo sapiens lipocalin 2 (LCN2), mRNA [NM_005564] |
| 19.490452 | SPRR2A | Homo sapiens small proline-rich protein 2A (SPRR2A), mRNA [NM_005988] |
| 19.353662 | DEFB1 | Homo sapiens defensin, beta 1 (DEFB1), mRNA [NM_005218] |
| 18.580254 | SMIM5 | Homo sapiens small integral membrane protein 5 (SMIM5), transcript variant 1, mRNA [NM_001162995] |
| 17.226309 | CNN1 | Homo sapiens calponin 1, basic, smooth muscle (CNN1), mRNA [NM_001299] |
| 16.54596 | SLPI | Homo sapiens secretory leukocyte peptidase inhibitor (SLPI), mRNA [NM_003064] |
| 15.258101 | TENM2 | Homo sapiens teneurin transmembrane protein 2 (TENM2), mRNA [NM_001122679] |
| 15.045251 | SMIM22 | Homo sapiens small integral membrane protein 22 (SMIM22), transcript variant 1, mRNA [NM_001253790] |
| 13.634027 | STRA6 | Homo sapiens stimulated by retinoic acid 6 (STRA6), transcript variant 8, mRNA [NM_001199042] |
| 13.483241 | KLK7 | Homo sapiens kallikrein-related peptidase 7 (KLK7), transcript variant 1, mRNA [NM_005046] |
| 13.081661 | IL6 | Homo sapiens interleukin 6 (interferon, beta 2) (IL6), mRNA [NM_000600] |
| 12.955583 | ANK3 | Homo sapiens ankyrin 3, node of Ranvier (ankyrin G) (ANK3), transcript variant 1, mRNA [NM_020987] |
| 12.833475 | FAM83A | Homo sapiens family with sequence similarity 83, member A (FAM83A), transcript variant 2, mRNA [NM_207006] |
| 12.62631 | GRB10 | Homo sapiens growth factor receptor-bound protein 10 (GRB10), transcript variant 4, mRNA [NM_001001555] |
| 12.033352 | TRIML2 | Homo sapiens tripartite motif family-like 2 (TRIML2), mRNA [NM_173553] |
| 11.569868 | DOCK11 | Homo sapiens dedicator of cytokinesis 11 (DOCK11), mRNA [NM_144658] |
| 11.453753 | IL32 | Homo sapiens interleukin 32 (IL32), transcript variant 1, mRNA [NM_001012631] |
| 11.449253 | ARC | Homo sapiens activity-regulated cytoskeleton-associated protein (ARC), mRNA [NM_015193] |
| 11.411928 | CXCL1 | Homo sapiens chemokine (C-X-C motif) ligand 1 (melanoma growth stimulating activity, alpha) (CXCL1), transcript variant 1, mRNA [NM_001511] |
| 11.275037 | GGTLC2 | Homo sapiens gamma-glutamyltransferase light chain 2 (GGTLC2), transcript variant 3, mRNA [NM_001282879] |
| 10.952953 | CXCL2 | Homo sapiens chemokine (C-X-C motif) ligand 2 (CXCL2), mRNA [NM_002089] |
| 10.904443 | CCL20 | Homo sapiens chemokine (C-C motif) ligand 20 (CCL20), transcript variant 1, mRNA [NM_004591] |
| 10.799985 | KRT42P | Homo sapiens keratin 42 pseudogene (KRT42P), non-coding RNA [NR_033415] |
| 10.332065 | SPRR2E | Homo sapiens small proline-rich protein 2E (SPRR2E), mRNA [NM_001024209] |
| 10.251256 | SUN3 | Homo sapiens Sad1 and UNC84 domain containing 3 (SUN3), transcript variant 1, mRNA [NM_001030019] |
| 10.21648 | RFTN1 | Homo sapiens raftlin, lipid raft linker 1 (RFTN1), mRNA [NM_015150] |
| 9.594469 | CCL3 | Homo sapiens chemokine (C-C motif) ligand 3 (CCL3), mRNA [NM_002983] |
| 9.552788 | CXCL3 | Homo sapiens chemokine (C-X-C motif) ligand 3 (CXCL3), mRNA [NM_002090] |
| 9.534228 | CARD16 | Homo sapiens caspase recruitment domain family, member 16 (CARD16), transcript variant 1, mRNA [NM_001017534] |
| 9.448945 | PDZK1IP1 | Homo sapiens PDZK1 interacting protein 1 (PDZK1IP1), mRNA [NM_005764] |
| 9.195005 | KLK11 | Homo sapiens kallikrein-related peptidase 11 (KLK11), transcript variant 2, mRNA [NM_144947] |
| 9.159128 | C16orf54 | Homo sapiens chromosome 16 open reading frame 54 (C16orf54), mRNA [NM_175900] |
| 8.975004 | GGT6 | Homo sapiens gamma-glutamyltransferase 6 (GGT6), transcript variant 2, mRNA [NM_153338] |
| 8.824032 | CD38 | Homo sapiens CD38 molecule (CD38), mRNA [NM_001775] |
| 8.594149 | LINC00640 | Homo sapiens long intergenic non-protein coding RNA 640 (LINC00640), long non-coding RNA [NR_038358] |
| 8.430595 | LOC645638 |  |
| 8.382051 | CAGE1 | Homo sapiens cancer antigen 1 (CAGE1), transcript variant 1, mRNA [NM_001170692] |
| 8.32493 | C3 | Homo sapiens complement component 3 (C3), mRNA [NM_000064] |
| 8.267075 | IL1A | Homo sapiens interleukin 1, alpha (IL1A), mRNA [NM_000575] |
| 8.208855 | PCSK1 | Homo sapiens proprotein convertase subtilisin/kexin type 1 (PCSK1), transcript variant 1, mRNA [NM_000439] |
| 8.003435 | IL20RB | Homo sapiens interleukin 20 receptor beta (IL20RB), mRNA [NM_144717] |
| 7.889108 | GGTLC1 | Homo sapiens gamma-glutamyltransferase light chain 1 (GGTLC1), transcript variant A, mRNA [NM_178311] |
| 7.859583 | PPP4R4 | Homo sapiens protein phosphatase 4, regulatory subunit 4 (PPP4R4), transcript variant 2, mRNA [NM_020958] |
| 7.834644 | TRIQK | Homo sapiens triple QxxK/R motif containing (TRIQK), transcript variant 1, mRNA [NM_001171796] |
| 7.7930083 | KLHL30 | Homo sapiens kelch-like family member 30 (KLHL30), mRNA [NM_198582] |
| 7.7170734 | KRT14 | Homo sapiens keratin 14 (KRT14), mRNA [NM_000526] |
| 7.6150427 | KRT17 | Homo sapiens keratin 17 (KRT17), mRNA [NM_000422] |
| 7.565798 | CREB5 | Homo sapiens cAMP responsive element binding protein 5 (CREB5), transcript variant 1, mRNA [NM_182898] |
| 7.561192 | CCDC3 | Homo sapiens coiled-coil domain containing 3 (CCDC3), transcript variant 1, mRNA [NM_031455] |
| 7.5277467 | SORCS2 | Homo sapiens sortilin-related VPS10 domain containing receptor 2 (SORCS2), mRNA [NM_020777] |
| 7.481977 | LOC101929942 | PREDICTED: Homo sapiens uncharacterized LOC101929942 (LOC101929942), transcript variant X3, misc_RNA [XR_252326] |
| 7.3587055 | ARHGAP25 | Homo sapiens Rho GTPase activating protein 25 (ARHGAP25), transcript variant 1, mRNA [NM_001007231] |
| 7.3491554 | CSGALNACT1 | Homo sapiens chondroitin sulfate N-acetylgalactosaminyltransferase 1 (CSGALNACT1), transcript variant 2, mRNA [NM_018371] |
| 7.3420434 | PLEKHA6 | Homo sapiens pleckstrin homology domain containing, family A member 6 (PLEKHA6), mRNA [NM_014935] |
| 7.2742567 | CTSO | Homo sapiens cathepsin O (CTSO), mRNA [NM_001334] |
| 7.2261105 | WIPI1 | Homo sapiens WD repeat domain, phosphoinositide interacting 1 (WIPI1), mRNA [NM_017983] |
| 7.188539 | INHBA | Homo sapiens inhibin, beta A (INHBA), mRNA [NM_002192] |
| 7.1044874 | DNAH9 | Homo sapiens dynein, axonemal, heavy chain 9 (DNAH9), transcript variant 2, mRNA [NM_001372] |
| 7.096404 | ANO2 | Homo sapiens anoctamin 2 (ANO2), transcript variant 2, mRNA [NM_001278597] |
| 7.03618 | THEG | Homo sapiens theg spermatid protein (THEG), transcript variant 1, mRNA [NM_016585] |
| 7.01556 | NLRP7 | Homo sapiens NLR family, pyrin domain containing 7 (NLRP7), transcript variant 1, mRNA [NM_139176] |
| 6.9046683 | MAOA | Homo sapiens monoamine oxidase A (MAOA), transcript variant 2, mRNA [NM_001270458] |
| 6.893099 | IL8 | Homo sapiens interleukin 8 (IL8), mRNA [NM_000584] |
| 6.8257103 | IL36G | Homo sapiens interleukin 36, gamma (IL36G), transcript variant 1, mRNA [NM_019618] |
| 6.4689007 | APOL3 | Homo sapiens apolipoprotein L, 3 (APOL3), transcript variant beta/a, mRNA [NM_145641] |
| 6.427765 | THAP9-AS1 | Homo sapiens THAP9 antisense RNA 1 (THAP9-AS1), transcript variant 1, long non-coding RNA [NR_034075] |
| 6.4252605 | HBEGF | Homo sapiens heparin-binding EGF-like growth factor (HBEGF), mRNA [NM_001945] |
| 6.3742666 | SEZ6L2 | Homo sapiens seizure related 6 homolog (mouse)-like 2 (SEZ6L2), transcript variant 2, mRNA [NM_201575] |
| 6.326575 | C1S | Homo sapiens complement component 1, s subcomponent (C1S), transcript variant 2, mRNA [NM_001734] |
| 6.2491794 | COL13A1 | Homo sapiens collagen, type XIII, alpha 1 (COL13A1), transcript variant 5, mRNA [NM_080801] |
| 6.2177706 | CFB | Homo sapiens complement factor B (CFB), mRNA [NM_001710] |
| 6.2124047 | SIMC1 | Homo sapiens SUMO-interacting motifs containing 1 (SIMC1), mRNA [NM_198567] |
| 6.186055 | CSF2 | Homo sapiens colony stimulating factor 2 (granulocyte-macrophage) (CSF2), mRNA [NM_000758] |
| 6.0473294 | SYTL3 | Homo sapiens synaptotagmin-like 3 (SYTL3), transcript variant 3, mRNA [NM_001009991] |
| 5.9935794 | RHBDL2 | Homo sapiens rhomboid, veinlet-like 2 (Drosophila) (RHBDL2), mRNA [NM_017821] |
| 5.977982 | HLA-DQB1 | Homo sapiens major histocompatibility complex, class II, DQ beta 1 (HLA-DQB1), transcript variant 3, mRNA [NM_001243962] |
| 5.973145 | TGM1 | Homo sapiens transglutaminase 1 (TGM1), mRNA [NM_000359] |
| 5.964998 | HMSD | Homo sapiens histocompatibility (minor) serpin domain containing (HMSD), mRNA [NM_001123366] |
| 5.9413 | ZBED2 | Homo sapiens zinc finger, BED-type containing 2 (ZBED2), mRNA [NM_024508] |
| 5.9144053 | S100A7 | Homo sapiens S100 calcium binding protein A7 (S100A7), mRNA [NM_002963] |
| 5.8909926 | KCNJ5 | Homo sapiens potassium inwardly-rectifying channel, subfamily J, member 5 (KCNJ5), mRNA [NM_000890] |
| 5.8478475 | LPAR5 | Homo sapiens lysophosphatidic acid receptor 5 (LPAR5), transcript variant 1, mRNA [NM_020400] |
| 5.8378787 | BMPER | Homo sapiens BMP binding endothelial regulator (BMPER), mRNA [NM_133468] |
| 5.7913074 | ZNF713 | Homo sapiens zinc finger protein 713 (ZNF713), mRNA [NM_182633] |
| 5.7241063 | CDK14 | Homo sapiens cyclin-dependent kinase 14 (CDK14), mRNA [NM_012395] |
| 5.583558 | DNER | Homo sapiens delta/notch-like EGF repeat containing (DNER), mRNA [NM_139072] |
| 5.4782634 | SAA4 | Homo sapiens serum amyloid A4, constitutive (SAA4), mRNA [NM_006512] |
| 5.4427733 | KIAA1407 | Homo sapiens KIAA1407 (KIAA1407), mRNA [NM_020817] |
| 5.413598 | FOXQ1 | Homo sapiens forkhead box Q1 (FOXQ1), mRNA [NM_033260] |
| 5.355569 | LOC400743 | Homo sapiens cDNA FLJ45933 fis, clone PLACE7003639. [AK127830] |
| 5.282676 | PIP5KL1 | Homo sapiens phosphatidylinositol-4-phosphate 5-kinase-like 1 (PIP5KL1), transcript variant 1, mRNA [NM_001135219] |
| 5.2673073 | ITGBL1 | Homo sapiens integrin, beta-like 1 (with EGF-like repeat domains) (ITGBL1), transcript variant 1, mRNA [NM_004791] |
| 5.262269 | LOC100130691 | Homo sapiens uncharacterized LOC100130691 (LOC100130691), long non-coding RNA [NR_026966] |
| 5.261373 | LCK | Homo sapiens lymphocyte-specific protein tyrosine kinase (LCK), transcript variant 2, mRNA [NM_005356] |
| 5.234131 | LOC100505633 | Homo sapiens uncharacterized LOC100505633 (LOC100505633), long non-coding RNA [NR_038849] |
| 5.2238092 | KRT16P2 | Homo sapiens keratin 16 pseudogene 2 (KRT16P2), non-coding RNA [NR_029392] |
| 5.2017293 | UBD | Homo sapiens ubiquitin D (UBD), mRNA [NM_006398] |
| 5.1961226 | SERPINB3 | Homo sapiens serpin peptidase inhibitor, clade B (ovalbumin), member 3 (SERPINB3), mRNA [NM_006919] |
| 5.136296 | MGLL | Homo sapiens monoglyceride lipase (MGLL), transcript variant 1, mRNA [NM_007283] |
| 5.0818067 | LOC101930067 | PREDICTED: Homo sapiens uncharacterized LOC101930067 (LOC101930067), misc_RNA [XR_250705] |
| 5.075375 | MGC50722 | Homo sapiens uncharacterized MGC50722 (MGC50722), mRNA [NM_203348] |
| 5.059058 | BMP2 | Homo sapiens bone morphogenetic protein 2 (BMP2), mRNA [NM_001200] |
| 5.0234346 | IGFL1 | Homo sapiens IGF-like family member 1 (IGFL1), mRNA [NM_198541] |
| 5.0080633 | S100P | Homo sapiens S100 calcium binding protein P (S100P), mRNA [NM_005980] |
| 5.004286 | HLA-DRB5 | Homo sapiens major histocompatibility complex, class II, DR beta 5 (HLA-DRB5), mRNA [NM_002125] |
| 4.9966955 | BARX2 | Homo sapiens BARX homeobox 2 (BARX2), mRNA [NM_003658] |
| 4.9905205 | LEPR | Homo sapiens leptin receptor (LEPR), transcript variant 3, mRNA [NM_001003679] |
| 4.973739 | SERPINB4 | Homo sapiens serpin peptidase inhibitor, clade B (ovalbumin), member 4 (SERPINB4), mRNA [NM_002974] |
| 4.919111 | RAET1E | Homo sapiens retinoic acid early transcript 1E (RAET1E), transcript variant 1, mRNA [NM_139165] |
| 4.864244 | SLC22A1 | Homo sapiens solute carrier family 22 (organic cation transporter), member 1 (SLC22A1), transcript variant 2, mRNA [NM_153187] |
| 4.846962 | LOC100130476 | Homo sapiens uncharacterized LOC100130476 (LOC100130476), long non-coding RNA [NR_049793] |
| 4.7776713 | JUN | Homo sapiens jun proto-oncogene (JUN), mRNA [NM_002228] |
| 4.7520123 | EMP3 | Homo sapiens epithelial membrane protein 3 (EMP3), mRNA [NM_001425] |
| 4.740523 | PTPRR | Homo sapiens protein tyrosine phosphatase, receptor type, R (PTPRR), transcript variant 1, mRNA [NM_002849] |
| 4.6810102 | ALDOC | Homo sapiens aldolase C, fructose-bisphosphate (ALDOC), mRNA [NM_005165] |
| 4.620012 | IL11 | Homo sapiens interleukin 11 (IL11), transcript variant 1, mRNA [NM_000641] |
| 4.613473 | GATM | Homo sapiens glycine amidinotransferase (L-arginine:glycine amidinotransferase) (GATM), mRNA [NM_001482] |
| 4.6124554 | KRT75 | Homo sapiens keratin 75 (KRT75), mRNA [NM_004693] |
| 4.607534 | KRT34 | Homo sapiens keratin 34 (KRT34), mRNA [NM_021013] |
| 4.588996 | LURAP1L | Homo sapiens leucine rich adaptor protein 1-like (LURAP1L), mRNA [NM_203403] |
| 4.5742044 | KCNJ12 | Homo sapiens potassium inwardly-rectifying channel, subfamily J, member 12 (KCNJ12), mRNA [NM_021012] |
| 4.571703 | GPATCH4 | G patch domain containing 4 [Source:HGNC Symbol;Acc:25982] [ENST00000334588] |
| 4.505633 | RPRD1A | Homo sapiens regulation of nuclear pre-mRNA domain containing 1A (RPRD1A), mRNA [NM_018170] |
| 4.480093 | SOX9-AS1 | Homo sapiens SOX9 antisense RNA 1 (SOX9-AS1), transcript variant 1, long non-coding RNA [NR_103738] |
| 4.475948 | CLIC3 | Homo sapiens chloride intracellular channel 3 (CLIC3), mRNA [NM_004669] |
| 4.4272604 | NEDD4L | Homo sapiens neural precursor cell expressed, developmentally down-regulated 4-like, E3 ubiquitin protein ligase (NEDD4L), transcript variant j, mRNA [NM_001144967] |
| 4.36687 | TP53AIP1 | Homo sapiens tumor protein p53 regulated apoptosis inducing protein 1 (TP53AIP1), transcript variant 2, mRNA [NM_001195195] |
| 4.3562737 | ADAM21 | Homo sapiens ADAM metallopeptidase domain 21 (ADAM21), mRNA [NM_003813] |
| 4.3359346 | LINC00886 | Homo sapiens long intergenic non-protein coding RNA 886 (LINC00886), long non-coding RNA [NR_038387] |
| 4.2832837 | STAT5A | Homo sapiens signal transducer and activator of transcription 5A (STAT5A), mRNA [NM_003152] |
| 4.2593 | TNF | Homo sapiens tumor necrosis factor (TNF), mRNA [NM_000594] |
| 4.2298822 | ETV5 | Homo sapiens ets variant 5 (ETV5), mRNA [NM_004454] |
| 4.22098 | NEURL3 | Homo sapiens neuralized E3 ubiquitin protein ligase 3 (NEURL3), transcript variant 2, mRNA [NM_001285486] |
| 4.204461 | RELB | Homo sapiens v-rel avian reticuloendotheliosis viral oncogene homolog B (RELB), mRNA [NM_006509] |
| 4.203099 | ABCA1 | Homo sapiens ATP-binding cassette, sub-family A (ABC1), member 1 (ABCA1), mRNA [NM_005502] |
| 4.2016535 | VSTM1 | Homo sapiens V-set and transmembrane domain containing 1 (VSTM1), mRNA [NM_198481] |
| 4.1788316 | GGT1 | Homo sapiens gamma-glutamyltransferase 1 (GGT1), transcript variant 1, mRNA [NM_005265] |
| 4.1452236 | FOSB | Homo sapiens FBJ murine osteosarcoma viral oncogene homolog B (FOSB), transcript variant 1, mRNA [NM_006732] |
| 4.138653 | ARHGAP42 | Homo sapiens Rho GTPase activating protein 42 (ARHGAP42), mRNA [NM_152432] |
| 4.1350513 | VNN1 | Homo sapiens vanin 1 (VNN1), mRNA [NM_004666] |
| 4.066708 | GJB4 | Homo sapiens gap junction protein, beta 4, 30.3kDa (GJB4), mRNA [NM_153212] |
| 4.046508 | ECM2 | Homo sapiens extracellular matrix protein 2, female organ and adipocyte specific (ECM2), transcript variant 1, mRNA [NM_001393] |
| 4.0335503 | PCSK9 | Homo sapiens proprotein convertase subtilisin/kexin type 9 (PCSK9), mRNA [NM_174936] |
| 3.9825993 | HDAC9 | Homo sapiens histone deacetylase 9 (HDAC9), transcript variant 3, mRNA [NM_014707] |
| 3.971317 | KBTBD11 | Homo sapiens kelch repeat and BTB (POZ) domain containing 11 (KBTBD11), mRNA [NM_014867] |
| 3.96402 | ARHGAP26 | Homo sapiens Rho GTPase activating protein 26 (ARHGAP26), transcript variant 1, mRNA [NM_015071] |
| 3.9632032 | TRAF1 | Homo sapiens TNF receptor-associated factor 1 (TRAF1), transcript variant 1, mRNA [NM_005658] |
| 3.9557018 | EHF | Homo sapiens ets homologous factor (EHF), transcript variant 2, mRNA [NM_012153] |
| 3.9542854 | MGAT4A | Homo sapiens mannosyl (alpha-1,3-)-glycoprotein beta-1,4-N-acetylglucosaminyltransferase, isozyme A (MGAT4A), transcript variant 1, mRNA [NM_012214] |
| 3.9050076 | CHODL | Homo sapiens chondrolectin (CHODL), transcript variant 1, mRNA [NM_024944] |
| 3.8764012 | GGT3P | Homo sapiens gamma-glutamyltransferase 3 pseudogene (GGT3P), non-coding RNA [NR_003267] |
| 3.8393097 | NNMT | Homo sapiens nicotinamide N-methyltransferase (NNMT), mRNA [NM_006169] |
| 3.8305998 | KYNU | Homo sapiens kynureninase (KYNU), transcript variant 2, mRNA [NM_001032998] |
| 3.8160796 | XDH | Homo sapiens xanthine dehydrogenase (XDH), mRNA [NM_000379] |
| 3.7800643 | FAM228B | Homo sapiens family with sequence similarity 228, member B (FAM228B), mRNA [NM_001145710] |
| 3.7735002 | SPRR2D | Homo sapiens small proline-rich protein 2D (SPRR2D), mRNA [NM_006945] |
| 3.771482 | MCAM | Homo sapiens melanoma cell adhesion molecule (MCAM), mRNA [NM_006500] |
| 3.7617493 | TEF | Homo sapiens thyrotrophic embryonic factor (TEF), transcript variant 1, mRNA [NM_003216] |
| 3.751711 | LY96 | Homo sapiens lymphocyte antigen 96 (LY96), transcript variant 1, mRNA [NM_015364] |
| 3.7434523 | ABLIM3 | Homo sapiens actin binding LIM protein family, member 3 (ABLIM3), mRNA [NM_014945] |
| 3.7298934 | NPAS2 | Homo sapiens neuronal PAS domain protein 2 (NPAS2), mRNA [NM_002518] |
| 3.7042816 | MAP3K8 | Homo sapiens mitogen-activated protein kinase kinase kinase 8 (MAP3K8), transcript variant 1, mRNA [NM_005204] |
| 3.6764019 | NR1D2 | Homo sapiens nuclear receptor subfamily 1, group D, member 2 (NR1D2), transcript variant 1, mRNA [NM_005126] |
| 3.6694694 | YAE1D1 | Homo sapiens Yae1 domain containing 1 (YAE1D1), transcript variant 2, mRNA [NM_001282446] |
| 3.6684792 | GUCY1B2 | Homo sapiens guanylate cyclase 1, soluble, beta 2 (pseudogene) (GUCY1B2), non-coding RNA [NR_003923] |
| 3.667418 | KRT83 | Homo sapiens keratin 83 (KRT83), mRNA [NM_002282] |
| 3.654501 | SMPDL3B | Homo sapiens sphingomyelin phosphodiesterase, acid-like 3B (SMPDL3B), transcript variant 2, mRNA [NM_001009568] |
| 3.6454031 | GNG11 | Homo sapiens guanine nucleotide binding protein (G protein), gamma 11 (GNG11), mRNA [NM_004126] |
| 3.6385214 | IL1B | Homo sapiens interleukin 1, beta (IL1B), mRNA [NM_000576] |
| 3.6383915 | GRHL3 | Homo sapiens grainyhead-like 3 (Drosophila) (GRHL3), transcript variant 2, mRNA [NM_198173] |
| 3.637877 | KRT6C | Homo sapiens keratin 6C (KRT6C), mRNA [NM_173086] |
| 3.6225488 | CAPNS2 | Homo sapiens calpain, small subunit 2 (CAPNS2), mRNA [NM_032330] |
| 3.6041224 | PLAU | Homo sapiens plasminogen activator, urokinase (PLAU), transcript variant 1, mRNA [NM_002658] |
| 3.6005325 | BATF2 | Homo sapiens basic leucine zipper transcription factor, ATF-like 2 (BATF2), mRNA [NM_138456] |
| 3.5832307 | ACER3 | Homo sapiens alkaline ceramidase 3 (ACER3), mRNA [NM_018367] |
| 3.5775528 | BIRC3 | Homo sapiens baculoviral IAP repeat containing 3 (BIRC3), transcript variant 1, mRNA [NM_001165] |
| 3.5380442 | C12orf54 | Homo sapiens chromosome 12 open reading frame 54 (C12orf54), mRNA [NM_152319] |
| 3.5328908 | COL12A1 | Homo sapiens collagen, type XII, alpha 1 (COL12A1), transcript variant long, mRNA [NM_004370] |
| 3.5276303 | KRT6A | Homo sapiens keratin 6A (KRT6A), mRNA [NM_005554] |
| 3.507554 | ZG16B | Homo sapiens zymogen granule protein 16B (ZG16B), mRNA [NM_145252] |
| 3.5006578 | ZNF812 | Homo sapiens zinc finger protein 812 (ZNF812), mRNA [NM_001199814] |
| 3.4984543 | MYBPHL | Homo sapiens myosin binding protein H-like (MYBPHL), transcript variant 1, mRNA [NM_001010985] |
| 3.49609 | PDZK1 | Homo sapiens PDZ domain containing 1 (PDZK1), transcript variant 1, mRNA [NM_002614] |
| 3.480152 | ATP8B3 | Homo sapiens ATPase, aminophospholipid transporter, class I, type 8B, member 3 (ATP8B3), transcript variant 1, mRNA [NM_138813] |
| 3.4666378 | OTOP1 | Homo sapiens otopetrin 1 (OTOP1), mRNA [NM_177998] |
| 3.463664 | ATP8A2 | Homo sapiens ATPase, aminophospholipid transporter, class I, type 8A, member 2 (ATP8A2), mRNA [NM_016529] |
| 3.420165 | FLJ13744 | Homo sapiens cDNA FLJ13744 fis, clone PLACE3000230. [AK023806] |
| 3.405729 | SERPINB8 | Homo sapiens serpin peptidase inhibitor, clade B (ovalbumin), member 8 (SERPINB8), transcript variant 3, mRNA [NM_001031848] |
| 3.3920388 | ASCL2 | Homo sapiens achaete-scute family bHLH transcription factor 2 (ASCL2), mRNA [NM_005170] |
| 3.3824444 | MTSS1 | Homo sapiens metastasis suppressor 1 (MTSS1), transcript variant 1, mRNA [NM_001282971] |
| 3.3822398 | OGFRL1 | opioid growth factor receptor-like 1 [Source:HGNC Symbol;Acc:21378] [ENST00000370435] |
| 3.3193138 | LINC00663 | Homo sapiens long intergenic non-protein coding RNA 663 (LINC00663), long non-coding RNA [NR_026956] |
| 3.3072977 | LOC644662 | PREDICTED: Homo sapiens uncharacterized LOC644662 (LOC644662), misc_RNA [XR_171034] |
| 3.2947426 | PER1 | Homo sapiens period circadian clock 1 (PER1), mRNA [NM_002616] |
| 3.2923481 | ALMS1 | Homo sapiens Alstrom syndrome 1 (ALMS1), mRNA [NM_015120] |
| 3.2853425 | FAM87A | Homo sapiens family with sequence similarity 87, member A (FAM87A), long non-coding RNA [NR_103537] |
| 3.2620268 | DUSP1 | Homo sapiens dual specificity phosphatase 1 (DUSP1), mRNA [NM_004417] |
| 3.255715 | DDIT3 | Homo sapiens DNA-damage-inducible transcript 3 (DDIT3), transcript variant 5, mRNA [NM_004083] |
| 3.2512882 | FAAH2 | Homo sapiens fatty acid amide hydrolase 2 (FAAH2), mRNA [NM_174912] |
| 3.2484765 | GSAP | Homo sapiens gamma-secretase activating protein (GSAP), mRNA [NM_017439] |
| 3.2386491 | USH1G | Homo sapiens Usher syndrome 1G (autosomal recessive) (USH1G), transcript variant 1, mRNA [NM_173477] |
| 3.2220817 | USP53 | Homo sapiens ubiquitin specific peptidase 53 (USP53), mRNA [NM_019050] |
| 3.2091079 | ARHGEF17 | Homo sapiens Rho guanine nucleotide exchange factor (GEF) 17 (ARHGEF17), mRNA [NM_014786] |
| 3.1852167 | PRTFDC1 | Homo sapiens phosphoribosyl transferase domain containing 1 (PRTFDC1), transcript variant 1, mRNA [NM_020200] |
| 3.1801605 | LRRC56 | Homo sapiens leucine rich repeat containing 56 (LRRC56), mRNA [NM_198075] |
| 3.173223 | PARP11 | Homo sapiens poly (ADP-ribose) polymerase family, member 11 (PARP11), transcript variant 1, mRNA [NM_020367] |
| 3.1705737 | FAM83E | Homo sapiens family with sequence similarity 83, member E (FAM83E), mRNA [NM_017708] |
| 3.1643739 | ABCG1 | Homo sapiens ATP-binding cassette, sub-family G (WHITE), member 1 (ABCG1), transcript variant 5, mRNA [NM_207627] |
| 3.1586742 | ANTXR2 | Homo sapiens anthrax toxin receptor 2 (ANTXR2), transcript variant 1, mRNA [NM_058172] |
| 3.15758 | PHLDB2 | Homo sapiens pleckstrin homology-like domain, family B, member 2 (PHLDB2), transcript variant 1, mRNA [NM_001134438] |
| 3.1428852 | TNFAIP3 | Homo sapiens tumor necrosis factor, alpha-induced protein 3 (TNFAIP3), transcript variant 3, mRNA [NM_006290] |
| 3.1366537 | FOXB1 | Homo sapiens forkhead box B1 (FOXB1), mRNA [NM_012182] |
| 3.1327994 | ACTA2 | Homo sapiens actin, alpha 2, smooth muscle, aorta (ACTA2), transcript variant 2, mRNA [NM_001613] |
| 3.132552 | DDX60 | Homo sapiens DEAD (Asp-Glu-Ala-Asp) box polypeptide 60 (DDX60), mRNA [NM_017631] |
| 3.117678 | DUSP6 | Homo sapiens dual specificity phosphatase 6 (DUSP6), transcript variant 1, mRNA [NM_001946] |
| 3.1162677 | DUOX2 | Homo sapiens dual oxidase 2 (DUOX2), mRNA [NM_014080] |
| 3.1064072 | LOC730227 | Homo sapiens uncharacterized LOC730227 (LOC730227), transcript variant 1, long non-coding RNA [NR_034151] |
| 3.104846 | ATP2B1 | Homo sapiens ATPase, Ca++ transporting, plasma membrane 1 (ATP2B1), transcript variant 2, mRNA [NM_001682] |
| 3.099937 | SLC22A18AS | Homo sapiens solute carrier family 22 (organic cation transporter), member 18 antisense (SLC22A18AS), mRNA [NM_007105] |
| 3.094185 | MRAS | Homo sapiens muscle RAS oncogene homolog (MRAS), transcript variant 1, mRNA [NM_012219] |
| 3.09243 | DMBT1 | Homo sapiens deleted in malignant brain tumors 1 (DMBT1), transcript variant 2, mRNA [NM_007329] |
| 3.0904138 | BBS9 | Homo sapiens Bardet-Biedl syndrome 9 (BBS9), transcript variant 2, mRNA [NM_198428] |
| 3.0764973 | FEZ1 | Homo sapiens fasciculation and elongation protein zeta 1 (zygin I) (FEZ1), transcript variant 1, mRNA [NM_005103] |
| 3.0758786 | KISS1R | Homo sapiens KISS1 receptor (KISS1R), mRNA [NM_032551] |
| 3.0647774 | LEMD1 | Homo sapiens LEM domain containing 1 (LEMD1), transcript variant 3, mRNA [NM_001001552] |
| 3.05127 | PER2 | Homo sapiens period circadian clock 2 (PER2), mRNA [NM_022817] |
| 3.0483944 | SMIM13 | Homo sapiens small integral membrane protein 13 (SMIM13), mRNA [NM_001135575] |
| 3.042593 | LINC00909 | Homo sapiens long intergenic non-protein coding RNA 909 (LINC00909), long non-coding RNA [NR_024484] |
| 3.0414932 | LOXL4 | Homo sapiens lysyl oxidase-like 4 (LOXL4), mRNA [NM_032211] |
| 3.040714 | TRIM55 | Homo sapiens tripartite motif containing 55 (TRIM55), transcript variant 3, mRNA [NM_184086] |
| 3.0362651 | ITGAV | Homo sapiens integrin, alpha V (ITGAV), transcript variant 1, mRNA [NM_002210] |
| 3.0107121 | IL21R | Homo sapiens interleukin 21 receptor (IL21R), transcript variant 2, mRNA [NM_181078] |
| 3.004534 | LOC100506731 | PREDICTED: Homo sapiens uncharacterized LOC100506731 (LOC100506731), misc_RNA [XR_110231] |
